# Pharmacokinetics and Metabolism of 4R-Cembranoid

**DOI:** 10.1371/journal.pone.0121540

**Published:** 2015-03-26

**Authors:** Wanda Vélez-Carrasco, Carol E. Green, Paul Catz, Anna Furimsky, Kathleen O’Loughlin, Vesna A. Eterović, P. A. Ferchmin

**Affiliations:** 1 Department of Biochemistry, Universidad Central del Caribe, Bayamon, Puerto Rico, United States of America; 2 SRI International Biosciences Division, Menlo Park, California, United States of America; St Michael's Hospital, University of Toronto, CANADA

## Abstract

4R-cembranoid (4R) is a natural cyclic diterpenoid found in tobacco leaves that displays neuroprotective activity. 4R protects against NMDA, paraoxon (POX), and diisopropylfluorophosphate (DFP) damage in rat hippocampal slices and against DFP in rats *in vivo*. The purpose of this study was to examine the metabolism and pharmacokinetics of 4R as part of its preclinical development as a neuroprotective drug. 10 µM 4R was found to be very stable in plasma for up to 1 hr incubation. 4R metabolism in human microsomes was faster than in the rat. Ten metabolites of 4R were detected in the microsomal samples; 6 dihydroxy and 4 monohydroxy forms of 4R. Male rats received a single dose of 4R at 6 mg/kg i.v., i.m., or s.c. The i.v. group had the highest plasma concentration of 1017 ng/mL. The t_1/2_ was 36 min and reached the brain within 10 min. The brain peak concentration was 6516 ng/g. The peak plasma concentration in the i.m. group was 163 ng/mL compared to 138 ng/mL in the s.c. group. The t_1/2_ of 4R after i.m. and s.c. administration was approximately 1.5 hr. The brain peak concentration was 329 ng/g in the i.m. group and 323 ng/g for the s.c. group. The brain to plasma ratio in the i.v. group was 6.4, reached 10 min after dose, whereas in the i.m. and s.c. groups was 2.49 and 2.48, respectively, at 90 min after dose. Our data show that 4R crosses the BBB and concentrates in the brain where it exerts its neuroprotective effect.

## Introduction

**Fig 1:**
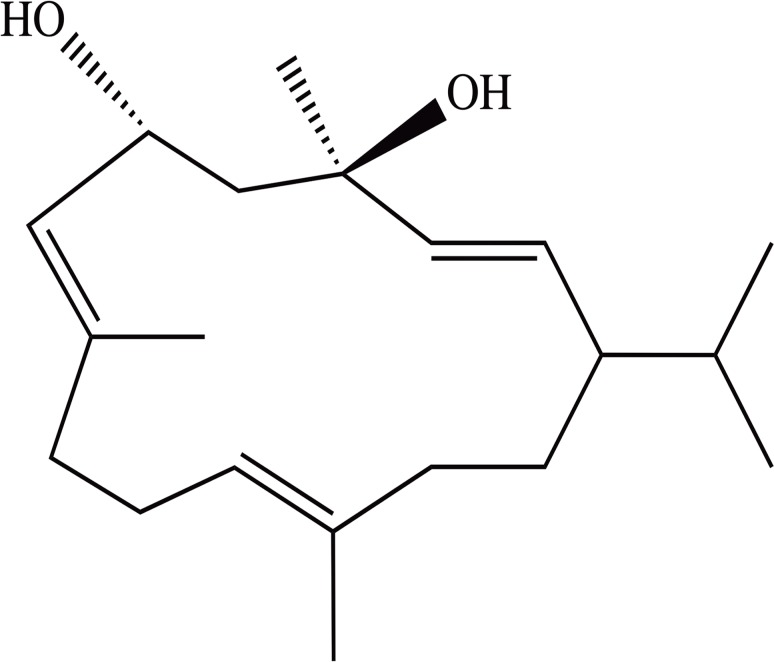
Structural formula of 4R.

4R-cembranoid [(1S, 2E, 4R, 6R, 7E, 11E)-cembra-2,7,11-triene-4,6-diol, abbreviated here as 4R] (Fig. 1) is a natural compound displaying neuroprotective and nicotine anti-addictive properties. Both activities involve 4R binding to nicotinic acetylcholine receptors (nAChR’s).

Cembranoids are natural diterpenoid compounds containing a 14-carbon cembrane ring substituted with oxygen-containing groups such as hydroxy, epoxy, oxo and lactone functions. Cembranoids are widespread in nature, found among other sources in soft corals and tobacco plants [1,2]. The two most abundant cembranoids in tobacco plants are 4R and its stereoisomer 4S [3].

*Ex vivo* studies using rat hippocampal slices reported that 4R protected neurons against N-methyl-D-aspartate (NMDA) neurotoxicity. Incubation of the slice with 4R before or after NMDA application prevented neuronal damage as measured by the capacity of neurons to elicit a population spike. 4R neuroprotection was not mediated by blocking NMDA receptors but involved activation of the PI3-kinase/Akt antiapoptotic signaling cascade. 4R-mediated activation of the PI-3 kinase/Akt antiapoptotic signaling pathway involves inhibition of the α7 nAChR and indirect activation of the α4β2 nAChRs [4,5].

4R has also been shown to protect neurons against organophosphate (OP) poisoning in rat hippocampal slices, and in vivo. Organophosphates are chemical compounds used as insecticides and as chemical warfare nerve agents. The neurotoxicity of OP’s is mainly due to the irreversible inhibition of acetylcholinesterase activity, this causes accumulation of acetylcholine and cholinergic overstimulation, which if untreated may be fatal. One example of OP’s is the insecticide parathion, whose active principle is the metabolite paraoxon (POX). Incubation of hippocampal slices with 4R before or after exposure to POX reversed the decrease in neuronal function observed with paraoxon alone [6]. Another OP compound, diisopropylfluorophosphate (DFP), is an analogue of the nerve agent sarin. Application of 4R after DFP exposure dramatically increased the population spike to almost the level observed in slices not exposed to DFP [7]. A concentration-protection curve showed a 50% population spike recovery with a 60nM 4R concentration. 4R administration subcutaneously to rats 1 hour before or 24 hr after DFP significantly decreased neuronal death and brain inflammation in the hippocampal area CA1 [8].

To our knowledge, there are no reports on the pharmacokinetics or metabolism of 4R. However, an in vitro study showed that 4R significantly increased the surface expression of the P-glycoprotein (P-gp) efflux transporter, which correlated with an increase in P-gp transport activity [9].

In addition to its role as a neuroprotective compound, 4R inhibited the expression of nicotine-induced behavioral sensitization in rats [10]. In another study, 4R exposure significantly decreased nicotine-induced withdrawal like behaviors in planarian worms [11]. Another set of studies demonstrated that 4R displays antitumor-promoting activities [12].

4R binds to and inhibits muscle-type and various neuronal-type nAChR’s [10]. 4R displaced the binding of the noncompetitive inhibitor [^3^H]-tenocyclidine (TCP) to the muscle-type nAChR from *T*.*californica* electric organ and inhibited in a noncompetitive manner the carbamoylcholine-induced ion flux in cells expressing human α4β2, α3β4, and muscle-type α1β1γδ nAChR’s. 4R was also shown to inhibit acetylcholine-induced currents in cells transfected with the human α7 nAChR [5].

The above described studies make 4R an attractive candidate for development as a therapeutic agent for neurodegenerative diseases and smoke-cessation therapy.

This current report is an initial 4R characterization study designed to determine the bioavailability, metabolism and pharmacokinetics of 4R after three different routes of administration to male rats.

## Materials and Methods

### Materials

Acetonitrile, methanol and formic acid were purchased from Mallinckrodt Baker, Inc. (Phillipsburg, NJ). Hexyl nicotinate was purchased from Sigma-Aldrich (Milwaukee, WI). Sprague-Dawley rat and human plasma was purchased from Bioreclamation Inc. (Hicksville, NY). Luna C18(2) HPLC columns (100 x 4.6 mm, 5 μm, and 50 x 2 mm, 5 μm) were purchased from Phenomenex Inc. (Torrance, CA).

### 4R Isolation

The cembranoid (1S, 2E, 4R, 6R, 7E, 11E)-cembra-2,7,11-triene-4,6-diol (4R) was prepared in the laboratory of Dr. K. El Sayed (School of Pharmacy, University of Louisiana, Monroe, LA) as previously described in [12]. The analytical criteria for characterization and purity were: 1) ^1^H NMR: integration of the H-6 proton at d 4.81 for 4R versus d 4.46 for the 4S epimer. 2) ^13^C NMR: C-6 in the 4R of d_c_ 67.5 versus C-6 in the 4S of d_c_ 66.0, and 3) thin-layer chromatography (TLC): R_f_ value of 0.42 (Si gel, *n*-hexane-ethyl acetate 1:1). The purity of 4R was more than 98%. 4R was prepared as a 10 mM stock in dimethyl sulfoxide (DMSO) and stored in aliquots at -20°C.

### In vitro metabolism and stability studies

#### 4R plasma stability

4R plasma stability was determined in vitro using rat and human plasma. The samples were incubated with 1 and 10 μM 4R for 0, 5, 15, 30, and 60 min at 37°C and for 60 min at room temperature. 4R was extracted with methanol to precipitate proteins, the supernatant evaporated to dryness, and reconstituted in 50/50/0.1 acetonitrile/water/formic acid (v/v/v) with 10 ng/mL hexyl nicotinate (internal standard). The samples were centrifuged at 18,000 x g for 10 min and the supernatants (100–250 μL) transferred to an HPLC vial containing 900 μl of 60% acetonitrile. The LC-MS/MS analysis was carried out using a high-performance liquid chromatography system consisting of a Shimadzu LC-20AD pump, (Shimadzu Scientific Instruments, Columbia, MA), and a CTC analytics PAL autosampler operating at 10°C (Leap technologies, Carrboro, NC). The MS instrument used was a 4000 QTrap triple quadrupole tandem mass spectrometer (Applied Biosystems/MDS Sciex, Foster City, CA). Electrospray ionization in positive ion mode (ESI+) was used. The detection of 4R was performed using the multiple reaction monitoring (MRM) mode. The mean percent 4R at each incubation time point was compared to the concentration at time 0. All samples were assayed in duplicate.

Calibration standards were prepared, (20, 70, 100, 200, 400, 700, and 1000 ng/mL) for plasma and brain homogenates. Quality Control (QC) samples (40, 500, and 900 ng/mL) were prepared using blank plasma and brain homogenates. The range of each calibration curve was 20.0 ng/mL (lower limit of quantitation, LLOQ) to 1000 ng/mL (upper limit of quantitation, ULOQ). Plasma samples above 1000 ng/mL were diluted 5-fold with blank matrix (rat plasma or rat brain homogenate) and a correction factor applied, so all measured values were within the calibration curve range. Quality control samples were included in the plasma sample analysis at 40.0 (low), 500 (medium), and 900 (high) ng/mL concentrations, and all of the QCs were within 90.81% of nominal, demonstrating appropriate accuracy. The precision of the QCs, as measured by relative standard deviation (%RSD), were within ± 7.98%, demonstrating appropriate precision of the method.

#### 4R metabolic stability

Pooled male and female human and rat liver microsomes were obtained commercially from BD Bioscience (San Jose, CA) and Xenotech (Lenexa, KS), respectively. Human and rat liver microsomes at 0.5 mg protein/mL concentration were incubated with 1 or 10 μM 4R and with cofactors (2.5 mM NADPH and 3.3 mM MgCl) in 100 mM phosphate buffer saline (PBS) pH 7.4 for 0, 5, 15, 30, and 60 min in a 37°C water bath. Reactions were started by the addition of microsomes and stopped by removing 100 μL aliquots at each time point. Each sample was mixed with acetonitrile containing hexyl nicotinate as internal standard, 1 μM 4R with 120 ng/mL and 10 μM 4R with 1200 ng/mL. As a negative control, microsomes were heat-inactivated for 10 min at 95°C and then incubated with 4R for 0 and 60 min. As positive control, 10μM midazolam was incubated with microsomes for 0, 15, 30 and 60 min in a 37°C water bath and with heat-inactivated microsomes for 0 and 60 min. The samples were analyzed for 4R using LC-MS/MS analysis described above. All assays were done in duplicates.

#### Identification of 4R metabolites

In order to detect major 4R metabolites, samples were prepared using the same protocol as for the metabolic stability studies, except that rat and human liver microsomes were incubated with 10 and 50 μM 4R in order to improve detection of 4R metabolites. As first negative control, 4R was incubated with heat-inactivated microsomes; as the second negative control, microsomes were incubated in the absence of 4R for 0 and 60 min to test for the presence of endogenous metabolites-like compounds. Reactions were stopped by removing 150 μL aliquots at 0, 5, 15, and 30 min and mixing with acetonitrile containing hexyl nicotinate as internal standard. A second aliquot of the extracted metabolite sample was obtained to determine the percent of 4R remaining. The samples were analyzed for 4R metabolites by LC-MS/MS. The LC method utilized a gradient elution with a total run time of 22 min, and allowed sufficient chromatographic separation of the detected metabolites. Samples were analyzed in full-scan mode with MS/MS product-ion spectra generated for detected metabolite masses. This was performed using the EMS (enhanced MS scan) mode of the mass spectrometer with IDA (independent data acquisition) experiments to produce MS/MS product-ion spectra for masses detected at sufficient intensities. The MS/MS spectra provided additional data to support the putative identities of the detected metabolites. All assays were done in duplicate.

### In vivo metabolism and pharmacokinetic studies

#### Formulations

The solubility of 4R at 12 mg/mL was tested using 5 different vehicle mixtures. The one with the best solubility at 12 mg/mL was chosen. 4R was prepared at room temperature by adding 10% DMSO and 90% polyethylene glycol 400 (PEG400). The 4R formulation was observed to be a clear colorless solution. The solution was sterile filtered using a Millipore Millex-GS 0.22 μm syringe filter. Dose formulations were prepared on Day 1 prior to dose administration and were maintained at room temperature until administration to the animals.

#### Rats

Twelve-week old male Sprague-Dawley rats weighing 307–373 g at the time of the experiment were used. The animals were randomly assigned to receive a single dose of 4R at 6 mg/kg by either i.v., i.m. or s.c. route. The dosing volume of injection was 0.5 mL/kg for the i.m. and s.c. routes and 5 mL/kg for the i.v. route. The i.v. 4R dose was administered via a jugular vein catheter and the s.c. dose in the distal scapular region. The i.m. 4R dose was given at the hind limb muscle site and the dose volume did not exceeded 0.1 mL in each i.m. site. Animal procedure for housing and care was performed according to the National Research Council (NRC) *Guide for the Care and Use of Laboratory Animals* and the Animal Welfare Standards incorporated in 9 Code of Federal Regulations Part 3, 1991. The animals had 12 hr light/dark cycles and at least 10 room volumes per hour with no recirculation of air. Purina certified rodent chow and water was provided ad libitum.

#### Clinical observations

Animals were examined for clinical signs related to the pharmacology and toxicology of 4R, gross motor and behavioral activity, and observable changes in appearance. The observations were done immediately post dose, and prior to last blood or brain collection.

#### Plasma and brain 4R concentrations

Blood samples were collected via a jugular vein catheter at each of the following time points: 2, 5, 10, 20, 45, 60, 90 min and 2, 4, 8 hr. 4R was extracted by adding 400 μL methanol to 50 μL of plasma. The samples were vortexed, centrifuged and the supernatants removed. The supernatants were evaporated to dryness and reconstituted in a solvent mixture containing 50/50/0.1 acetronile/water/formic acid (v/v/v) with 10ng/mL hexyl nicotinate as internal standard. After centrifugation at 14,000 x g for 5 min, 90 μL were transferred to an HPLC vial for 4R analysis by LC-MS/MS. Rat brains were collected at 10, 60 or 90 min, and 8 hr post dose and frozen at -70°C. On the day of the analysis, the brains were homogenized in PBS using 4 parts phosphate buffer and 1 part brain (g/g). 4R brain extraction was performed as described using plasma samples. The lower limit of quantitation of the LC-MS/MS was 20.0 ng/ml for plasma and 100 ng/g for brain.

#### Pharmacokinetic Analysis

Pharmacokinetic parameters for 4R were determined by subjecting data to non-compartmental analysis using WinNonlin Professional (v 6.2, Centara, St. Louis, MO) Model 201 (for i.v. administration) or 200 (for i.m. and s.c. administration); a uniform weighting factor was applied to each data set. Parameters were calculated for each individual animal. The peak concentration in plasma (C_max_) and the corresponding time of maximum concentration (T_max_) were determined directly from the data. The area under the plasma concentration-time curve (AUC) from time 0–8 hr was calculated using the log/linear trapezoidal (i.v. route) or linear up/log down trapezoidal (i.m. and s.c routes) methods. The dose administered was input to the program as mg/kg, and as a result no additional corrections for individual body weights of the animals were necessary. The following parameters and constants were determined for the three groups: observed maximum plasma concentration (C_max_), time to maximum plasma concentration (T_max_), area under the plasma concentration-time curve to the last time point (AUC_last_), area under the plasma concentration-time curve extrapolated to infinity (AUC_inf_), terminal phase elimination half-life (t_1/2_). Bioavailability (F) after i.m. and s.c. administration was calculated using their respective AUC_inf_ values divided by the AUC_inf_ value of the i.v. group. The ratio of 4R C_max_ between brain and plasma was calculated.

## Results

### In Vitro Stability and Metabolic Studies of 4R

The in vitro stability of 4R was determined after incubation of 1 and 10 μM 4R with rat and human plasma for 0, 15, 30, and 60 min at 37°C. With 10 μM 4R, the mean percent 4R content versus control ranged from 97–101% in rat plasma and from 96–103% in human plasma. After incubation for 60 min at room temperature, the mean percent 4R content was 96% and 98% in rat and human plasma, respectively. Similar results were obtained with 1 μM 4R (data not shown). These data indicate that 4R is stable in plasma up to a 60 min incubation period.

**Fig 2:**
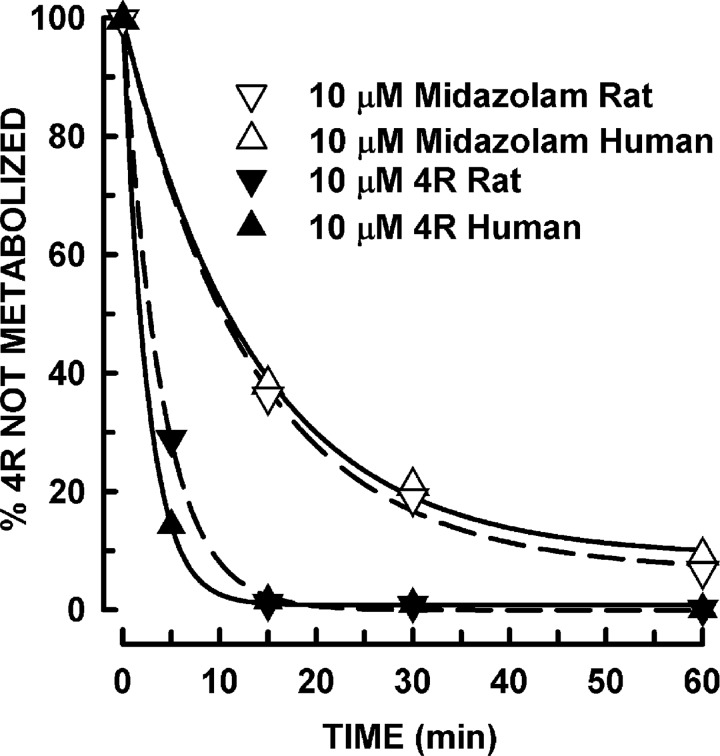
In vitro metabolic stability of 4R. 10 μM 4R was incubated with human and rat liver microsomes at selected time points (0, 5, 15, 30 and 60 min). As positive control, 10 μM midazolam was incubated with human and rat microsomes at 0, 15, 30 and 60 min. 4R levels were determined by LC-MS/MS. All samples were assayed in duplicate.

The metabolic stability of 4R was also tested in rat and human liver microsomes (Fig. 2). Midazolam (MDZ) was used as a positive control [13]. Incubation of microsomes with 10 μM MDZ showed the control being metabolized in both species. Incubation of MDZ with heat-inactivated microsomes showed no decrease in the percent remaining after 60 min incubation, human 89% and rat 108%. These results indicated that the conditions of the experiment were adequate. Ten micromolar 4R was rapidly metabolized by microsomes from both species. The metabolism of 4R in human microsomes was faster than that of the rat. In human microsomes, 15% remained after 5 min of incubation whereas 29% remained in rat microsomes. After 15 min of incubation, less than 2% of 4R remained in both species. Incubation of 4R with heat-inactivated microsomes showed no decrease in the percent of 4R remaining after 60 min of incubation, human 106% and rat 112%, which confirms 4R stability in the absence of metabolic activity.

Similar results were obtained when human and rat microsomes were incubated with 1 μM 4R. In human liver microsomes, 8% remained after 5 min of incubation whereas 19% remained in rat microsomes. After 15 min of incubation, 4% of 4R remained in both species. Incubation of 1 μM 4R with heat-inactivated microsomes showed no decrease in the percent of 4R remaining after 60 min of incubation in both species (human 108% and rat 136%).

For the detection of 4R metabolites, 10 and 50 μM 4R was incubated with rat and human microsomes. The percent 4R remaining in the 10 μM samples was similar to the 10 μM results from the metabolic stability experiment, demonstrating good reproducibility between experiments. In human microsomes, 18% remained after 5 min of incubation whereas 34% remained in rat microsomes. After 15 min of incubation, less than 2% of 4R remained in both species. As expected, the percent 4R remaining at 50 μM was higher compared to the 10 μM. However, there was still significant metabolism, as evidenced by ≤ 7.3% mean 4R remaining at 30 min in rat or human samples. At both 10 and 50 μM, 4R metabolism was faster in human compared to rat. Ten metabolites of 4R were detected in human and rat microsomal samples, four were mono-hydroxylated and six di-hydroxylated derivatives of 4R (Table 1). Upon ionization, 4R spontaneously dehydrates yielding a molecular ion of the form [M H2O +H]^+^. Thus, a mass at 289 m/z is the major form detected, instead of 307 m/z. A mass at 271 m/z (representing a second dehydration upon ionization) is also detected. The 289 m/z ion can then be subjected to collision-induced dissociation to yield 2 major fragment ions; 271 m/z (optimally forms at low collision energies), and 95 m/z (optimally forms at higher collision energies, i.e. 38eV). The ten fragment ions revealed a pattern of dehydration corresponding to mono (+16 amu) and di (+32 amu)-hydroxylated metabolites of 4R. These metabolites were not present in heat-inactivated human and rat microsomal samples incubated with 4R or in active microsomes without 4R. No other biotransformations, such as des-methyl, des-isopropyl, reductions, or combinations of biotransformations, were detected in the MRM mode, or in full-scan mode. These results do not preclude the possibility that other metabolites may be present but were not detected.

**Table 1:** Metabolites of 4R detected in 10 and 50 μM samples incubated with human and rat liver microsomes. Samples from human or rat liver microsomes incubated with 10 or 50 μM 4R generated the same metabolite species.

ID	RT^a^ (min)	Major Precursor Mass^b^ (m/z)	Precursor Form	Biotransformation
M1	6.29	303	[M-2H_2_O +H]^+^	di-hydroxylation
M2	7.02	303	[M-2H_2_O +H]^+^	di-hydroxylation
M3	7.93	321	[M-H_2_O +H]^+^	di-hydroxylation
M4	8.00	303	[M-2H_2_O +H]^+^	di-hydroxylation
M5	8.20	303	[M-2H_2_O +H]^+^	di-hydroxylation
M6	8.48	305	[M-H_2_O +H]^+^	hydroxylation
M7	8.76	287	[M-2H_2_O +H]^+^	hydroxylation
M8	9.11	321	[M-H_2_O +H]^+^	di-hydroxylation
M9	9.68	305	[M-H_2_O +H]^+^	hydroxylation
M10	9.92	305	[M-H_2_O +H]^+^	hydroxylation
4R	13.57	289	[M-H_2_O +H]^+^	(4R)

a. RT = retention time.

b. 4R metabolite masses were detected by LC-MS/MS in multiple reaction monitoring (MRM) mode using a range of precursor masses with a common product ion mass of 95 m/z, and a collision energy of 38 eV.

**Fig 3:**
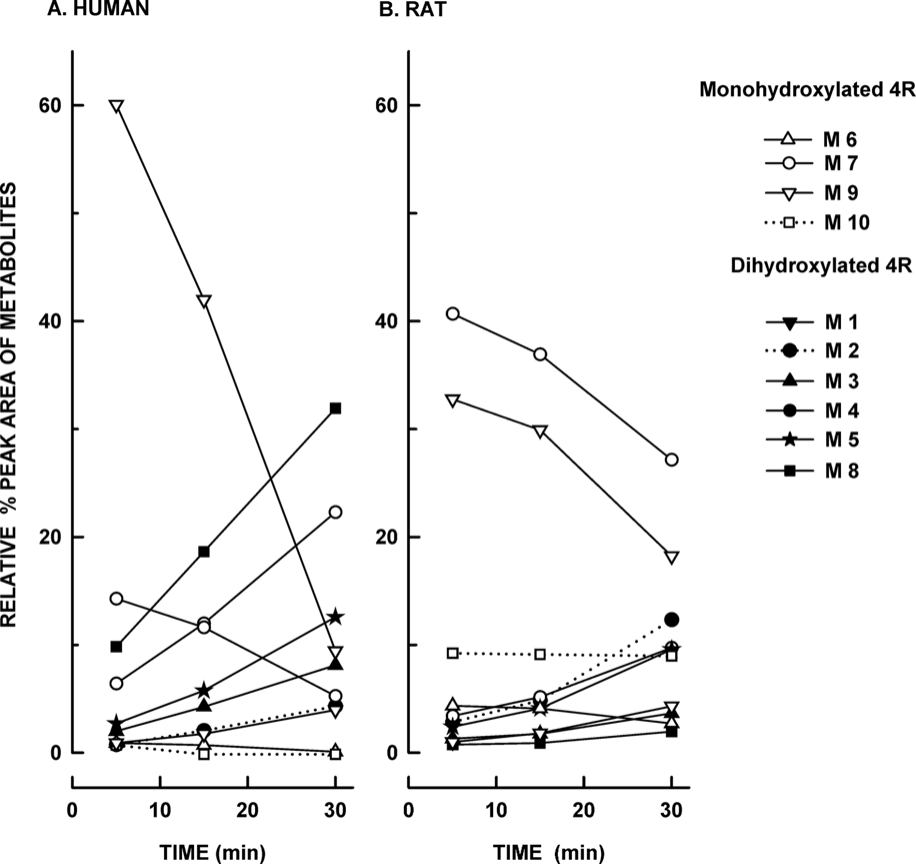
Relative percent of 4R metabolites in human (A) and rat (B) microsomal samples. 50 μM 4R microsomal human and rat samples were incubated for 5, 15, and 30 min. 4R metabolites levels were determined by LC-MS/MS. The symbols represent the relative percent peak area of each metabolite, which was calculated as the peak area of each metabolite per sample / total peak area of the 10 metabolites per sample * 100.

Fig. 3 shows the relative percent peak areas of the ten 4R metabolites in human (Fig. 3A) and rat (Fig. 3B) microsomal samples incubated for 5, 15, and 30 min. These results are not correlated to the percent remaining 4R in the samples and are not intended to be quantitative. The ionization efficiencies of the precursor and product ions are not known, and are not expected to be equal for each metabolite. With these caveats in mind, the major 4R metabolites detected in human samples were M9, M8, and M4. However, in the rat the major metabolites were M7 and M9, which indicates specie differences in the profile of 4R metabolites. The monohydroxylated 4R metabolites M9 and M7 reached their peak level within 5 min after incubation and then declined, possibly by conversion into the dihydroxylated metabolites. All dihydroxylated metabolites steadily increased during the 30 min incubation period. The peak areas of M6 and M10 metabolites did not change over time.

### In vivo plasma and brain 4R concentrations in male rats

Clinical observations revealed that rats given 4R by the i.m. and s.c. routes appeared normal. However, rats receiving 4R through the i.v. route had tremors and were hypoactive. All animals survived until the end of the study.

**Fig 4:**
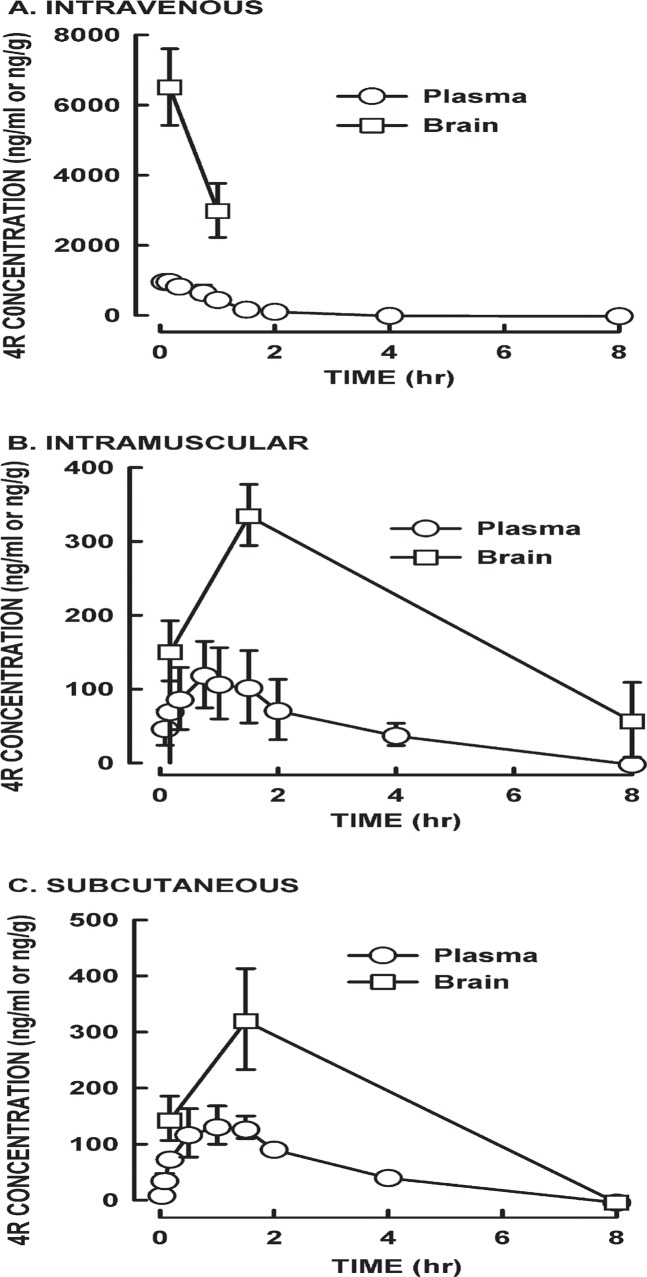
Plasma and brain 4R levels in male rats. Rats received 6 mg/kg of 4R by (A) i.v., (B) i.m., or (C) s.c. routes. Blood was collected at 2, 5, 10, 20, 45, 60, 90 min and 8 hr after administration. Brains were collected at 10 and 60 min after i.v. administration and at 10, 90 min and 8 hr after i.m. and s.c. administration. Samples were analyzed by LC-MS/MS for determination of 4R concentration. Values represent the mean ± SD of 3 rats.

Fig. 4 illustrates the time-concentration curves for the three administration routes. The time-plasma concentration curve for the i.v. group showed a mean plasma 4R concentration of 975 ± 104 ng/mL at 5 min, which steadily declined below the lower limit of quantitation (LLOQ< 20 ng/mL) by 8 hr post dose. The highest brain 4R levels were observed in the i.v. group, reaching a mean value of 6516 ±1091 ng/g at 10 min and decreasing to 2996 ±771 ng/g at 1 hr post dose.

For the i.m. and s.c. groups, the highest mean plasma 4R levels were 157 ± 86 ng/mL and 134 ± 34 ng/mL, respectively, and the time to maximum was 1 hr in both groups. Similar to the i.v. group, the plasma levels in the i.m. and s.c. groups were below the LLOQ by 8 hr post dose. Their maximum mean 4R brain levels were 336 ± 42 and 323 ± 90, respectively, and the time to maximum was 90 min. Their highest brain levels were approximately 20 fold lower than in the i.v. group. Brain 4R concentrations were higher than plasma in all three routes of administration. The i.v. group had the highest brain and plasma 4R concentrations at all time-points studied.

The plasma and brain samples were also analyzed to detect any of the mono or di-hydroxylated metabolites identified in the *in vitro* microsomal samples. Only trace levels were detected (≤ 3% relative peak area versus 4R).

### In vivo pharmacokinetic profile of 4R after administration to male rats

Table 2 summarizes the pharmacokinetic parameters of 4R given by the three different routes to rats. The i.v. group had the highest plasma peak concentration (C_max_) of 1017 ng/mL with a total 4R exposure over time (AUC_inf_) of 1141 ng.h/mL. The elimination t_1/2_ was fast (36 min) and reached the brain within 10 min post dose. The brain mean peak concentration was 6516 ng/g.

For the i.m. and s.c. groups, the kinetic results were very similar. The mean C_max_ plasma concentration in the i.m. group was 163 ng/mL compared to 138 ng/mL in the s.c. group at approximately 1 hr (T_max_) for both groups. The elimination t_1/2_ of 4R after i.m. and s.c. administration was approximately 1.5 hr. The mean AUC_inf_ values were 395 ng^.^h/mL and 466 ng^.^h/mL for the i.m. and s.c. groups, respectively. These values are much lower than the values observed in the i.v. group due to the lower bioavailability observed with the i.m. and s.c. routes. The fraction (F%) of 4R that reached circulation after i.m. and s.c. administration was 35% and 41%, respectively. The brain mean peak concentration was 329 ng/g in the i.m. group and 323 ng/g for the s.c. group. The C_max_ brain to plasma ratio in the i.v. group was 6.4, reached 10 min after dose, whereas in the i.m. and s.c. groups was 2.25 and 2.34, respectively, at 90 min after dose. These data shows that 4R crosses the BBB and concentrates in brain rapidly with i.v. injection and within 1.5 hr via i.m. and s.c. administration.

**Table 2:** Pharmacokinetic parameters of 4R in male rats. Rats received 6 mg/kg of 4R by either i.v., i.m. or s.c. routes. 4R levels were measured in plasma and brain samples at different time points. Values represent the mean ± SD. N = 3 rats for i.v. and s.c. and N = 6 for i.m.

Route	t_1/2_ (hr)	T_max_ (hr)	AUC_inf_ (hr.ng/mL	Plasma C_max_ (ng/mL)	Brain C_max_ (ng/g)	Ratio C_max_ brain/ plasma	F (%)
I.V.	0.6 ± 0.1	N/A	1141 ± 224	1017 ± 49	6516 ± 1091	6.41	100
I.M.	1.3 ± 0.4	1.0 ± 0.4	395 ± 138	146 ± 60	329 ± 50	2.25	35
S.C.	1.7 ± 0.1	1.2 ± 0.3	466 ± 56	138 ± 28	323 ± 90	2.34	41

t_½_, elimination half-life; T_max_, time to maximum plasma concentration; AUC_inf_, area under the plasma concentration-time curve extrapolated to infinity and C_max_, maximal plasma and brain concentration; F (%), bioavailability.

## Discussion

The cuticular layer of tobacco leaves contain cembranoids of which 4R is one of the most abundant. As mentioned in the introduction, 4R has been shown to neuroprotect against NMDA, paraoxon, and DFP neurotoxicity in vitro [4–7]. Furthermore, 4R administration subcutaneously to rats 1 hour before or 24 hr after DFP significantly decreased neuronal death in the hippocampal area CA1 [8]. The present study was designed to examine the metabolism and pharmacokinetics of 4R as part of its preclinical development.

Present data showed that 4R is rapidly metabolized in human liver microsomes in vitro. 4R metabolism involves hydroxylation of the 14-carbon ring to a more water soluble mono and dihydroxy metabolites. The monohydroxylated derivatives tended to decrease with time while the dihydroxylated increased suggesting a sequential hydroxylation. No other biotransformations, such as des-methyl, des-isopropyl, reductions, or combinations of biotransformations were detected. Parallel experiments with rat liver microsomes showed a similar pattern, but the metabolism was slower and the proportions of the various metabolites different. This effect may be due to differences in expression and catalytic activity of cytochrome p450 enzymes in humans versus rats [14].

Most biotransformations of chemical compounds are catalyzed by the cytochrome P450 proteins in almost all organisms [15].

Terpenes and cembranoids are catabolized by cytochrome P450 enzymes to hydroxylated metabolites in bacteria, insects and fish [16–18]. However, the breakdown of cembranoids in mammals, in particular 4R, has not been studied yet. Differential adaptation to dietary terpenoids was observed between generalist and specialist herbivores [19]. In the ringtail possum fed leaves of Eucalyptus radiata, the main excretion products of terpenes are polyoxygenated polar molecules that can readily be excreted. Minimal phase II biotransformations such as conjugation were detected [20].

In our study, midazolam was used as a rapidly metabolized positive control [13]. Interestingly, 4R was metabolized faster than midazolam. Therefore, it is unlikely that 4R will cause delayed toxic effects.

Incubation of 4R with human and rat plasma samples in vitro showed that 4R is stable in plasma for up to one hour. The stability in plasma is a requirement for compounds to be developed therapeutically unless they are pro-drugs which are expected to be modified in vivo to become active. Compounds that rapidly degrade in plasma often render misleading results in vitro, which are not applicable in vivo. In vivo pharmacokinetic studies and the storage and processing of samples containing such compounds are challenging. In addition, instability in plasma is in general a predictor of low bioavailability and poor therapeutic efficacy. Functional groups such as esters, lactones, amides, carbamides, lactams and sulphonamides are the characteristic of compounds susceptible to the hydrolysis by the plethora of plasma hydrolases. Considering the absence of such groups in 4R, it is not surprising that it is stable in plasma. The plasma stability of 4R facilitates the interpretation of data and suggests that 4R will show a reasonable bioavailability and therapeutic efficacy.

For the in vivo studies, rats were administered with a single dose of 6mg/kg 4R via three different routes. The i.v. and i.m. routes were chosen as likely methods of administration in future clinical uses for patients of nerve agents, where the oral route might not be practical. The s.c. route is convenient for efficacy determination in preclinical studies with rodents.

The distribution of 4R among brain and plasma injected either i.v., i.m., or s.c., showed consistently a fast penetration into brain and a higher concentration in brain than plasma at all times tested with the exception of 8 hours after s.c. injection when there was no detectable 4R. The 4R concentration in plasma decays very fast, probably by a combination of liver metabolism, absorption by various organs and excretion. The most important result for this work is the finding that 4R penetrates the blood-brain barrier very fast. This is a pre-requisite for the use of 4R as a neuroprotective agent in vivo. Relevant to 4R accumulation in the brain is the presence of drug efflux transporters in the BBB, which includes the P-glycoprotein (P-gp) efflux transporter, multidrug resistant proteins, and organic anion transporting polypeptides [21]. Interestingly, a study by Abzunit AH et al. [9] showed that 4R incubation increased the expression and efflux activity of the P-gp transporter in human colorectal cancer cells. Therefore, one possible explanation for the higher accumulation of 4R in brain after i.v. versus i.m. and s.c. administration could be the saturation of this transporter in the BBB.

The fast metabolism and elimination is also an advantage that minimizes the possibility of long-lasting permanence of 4R in brain that could cause untoward neurotoxicity. It could be argued that the rapid decay of 4R could decrease its therapeutic efficacy, but this could be easily solved by increasing the frequency of administration or using a slowly released 4R.

Only traces of metabolites were detected in plasma in vivo, possibly indicating fast excretion of these hydrophilic compounds. Lack of metabolites in the brain suggests that these compounds do not penetrate the BBB.

Clinical observations indicated that 6 mg/kg 4R s.c. or i.m., which resulted in brain C_max_ of 300–400 ng/mL did not produce any observable anomaly in the animals behavior or physiology. The same dose by i.v. route produced 20 times higher brain C_max_, which resulted in tremors and hypoactivity. All animals survived until the end of the experiment, 8 hr post dose. These observations indicate that 4R is a relatively save compound.

In conclusion, the results from this study indicate that 4R decays rapidly from blood and crosses the blood brain barrier where it concentrates and exerts its neuroprotective effect. Further in vivo studies need to address the specific localization and kinetics of 4R in brain.
